# Genetic and Non-genetic Factors Contributing to the Significant Variation in the Plasma Trough Concentration-to-Dose Ratio of Valproic Acid in Children With Epilepsy

**DOI:** 10.3389/fped.2020.599044

**Published:** 2021-01-20

**Authors:** Ze-Yue Xu, Hong-Li Guo, Ling Li, Min Zhang, Xia Jing, Ze-Jun Xu, Jin-Chun Qiu, Xiao-Peng Lu, Xuan-Sheng Ding, Feng Chen, Jing Xu

**Affiliations:** ^1^Department of Pharmacy, Children's Hospital of Nanjing Medical University, Nanjing, China; ^2^School of Basic Medicine and Clinical Pharmacy, China Pharmaceutical University, Nanjing, China; ^3^Department of Pharmacy, Boston Medical Center, Boston, MA, United States; ^4^Department of Neurology, Children's Hospital of Nanjing Medical University, Nanjing, China

**Keywords:** epilepsy, children, valproic acid, C0/D ratio, dosage form, therapeutic drug monitoring, polymorphism

## Abstract

**Objective:** This study was conducted to evaluate the potential genetic and non-genetic factors contributing to plasma trough concentration-to-dose (*C*_0_/*D*) ratio of valproic acid (VPA) in pediatric patients with epilepsy.

**Study Design:** A single-center, retrospective cohort study was performed by collecting data from 194 children aged 1–14 years between May 2018 and November 2018. The oral solution (*n* = 135) group and the sustained-release (SR) tablet group (*n* = 59) were defined, and the plasma VPA *C*_0_ was measured. Twenty-six single-nucleotide polymorphisms (SNPs) were chosen for genotyping with the MassARRAY system. A multiple logistic regression model was used for data analysis.

**Results:** Body weight (BW) and age were positively correlated with the *C*_0_/*D* ratio in 194 patients, but the positive correlation disappeared after the patients were divided into oral solution and SR tablet subgroups. The average *C*_0_/*D* ratio was significantly increased by 2.11-fold (*P* = 0.000) in children who took VPA SR tablets compared with children who were administered VPA oral solutions. No significant association between genetic variants and the *C*_0_/*D* ratio was found, even for the five well-studied SNPs, namely *UGT2B7* G211T, C802T, C161T, T125C, and CYP2C9^*^3 A1075C. However, a significant association between the *C*_0_/*D* ratio and *UGT1A6/9* Del>A (rs144486213) was observed in the VPA oral solution group, but not in the VPA SR tablet group.

**Conclusions:** The dosage forms of sodium valproate, rather than BW, age, or genetic polymorphisms, significantly affected the VPA *C*_0_/*D* ratios in pediatric patients with epilepsy. Based on our findings, switching the dosage form between solution and SR tablet should be performed cautiously. Total daily dose adjustment should be considered, and the plasma concentration, seizure-control effect, and adverse drug reaction should also be monitored very closely.

## Background

The incidence of epilepsy is age-dependent, and one of the highest incidence rates of epilepsy is observed in individuals aged <5 years (1). The incidence rate of epilepsy in children ranges from 41 to 187/100,000. The prevalence of epilepsy in children ranges from 3.2 to 5.5/1,000 in developed countries and 3.6 to 44/1,000 in underdeveloped countries, which are consistently higher than the incidence (1, 2). Children presenting with epilepsy before the age of 3 experience a high burden of cognitive and behavioral comorbidities (1). For most children with epilepsy, antiepileptic drugs (AEDs) are the main treatment modality to control, stop, or decrease the frequency of seizures as quickly as possible, leading to seizure freedom in ~70% of all children (3).

As a broad-spectrum antiepileptic drug, valproic acid (VPA, 2-propylpentanoic acid) has been widely used to treat almost all types of seizures and epilepsy syndromes (4). In particular, VPA remains the most effective drug for generalized seizures and is frequently used as a first-line agent for children with Lennox–Gastaut syndrome (3). Due to large interindividual differences in drug metabolism, the relationship between the VPA dose and plasma concentration is variable and inconsistent. The desired therapeutic effect is usually achieved within a reference range of plasma concentrations (50–100 μg/ml), with lower levels being more likely to produce an insufficient effect and higher levels being more frequently associated with adverse effects (5). Therefore, the unpredictable relationship between dose and VPA concentration supports the need to individualize and maintain the response based on therapeutic drug monitoring (TDM) (6).

Many factors including age, sex, total daily dose, formulations, genetic polymorphisms in drug-metabolizing enzymes, and transporters contribute to the individual variability in the systemic exposure levels of VPA. Multiple linear regression analysis showed that age and gender significantly affect the trough concentration of VPA (7). Another study suggested that older female patients require 30–50% lower doses of VPA than younger males (8). VPA undergoes extensive and complex hepatic metabolism (95%), with < 5% being excreted unchanged in the urine. Its hepatic metabolism occurs primarily via glucuronidation by UDP-glucuronosyltransferases (UGTs 1A3, 1A4, 1A6, 1A8, 1A9, 1A10, 2B7, and 2B15) and β-oxidation in the mitochondria. Valproate is also a substrate for cytochrome P450 (CYPs) 2C9 and 2C19, but these enzymes account for a relatively minor proportion of its elimination (9). Genetic polymorphisms in *UGTs*, including *UGT2B7* G211T (rs12233719) (7, 10), T802C (rs7439366) (7, 11, 12), and C161T (rs7668285) (10, 13), as well as *UGT1A6* A541G, and A552C (14), have been reported to be associated with the variable exposure level of VPA. A meta-analysis showed that *UGT2B7* variants G211T and C161T, but not T802C, affect the pharmacokinetics (PK) of VPA in patients with epilepsy (10). CYP-mediated ω-oxidation, a minor pathway of VPA metabolism in adults, appears to play an important role in the PK of VPA among pediatric patients (15–17). CYP2A6^*^4 and CYP2B6^*^6 increase systemic exposure to VPA (18). However, no significant correlation between CYP2A6^*^4 and VPA levels was observed in another study (19). Thus, the data described above are inconclusive.

VPA is available in a variety of formulations in clinical practice. Concerns related to treatment failure or the risk of adverse drug reactions have been raised by prescribers and patients around the time of switching between different products. Direct comparisons of PK showed that dose-adjusted extended-release (ER) formulations were not bioequivalent to their immediate-release (IR) counterparts for many AEDs, including carbamazepine, divalproate sodium, lamotrigine, oxcarbazepine, levetiracetam, and phenytoin (20). However, few studies focus on the effect of the dosage form on the variable *C*_0_/*D* ratios of VPA.

The objective of this study was to (1) summarize the TDM data from pediatric patients on VPA monotherapy; (2) evaluate the potential associations between *C*_0_/*D* ratio and age, BW, sex, and genetic polymorphisms in genes encoding UGTs and CYP450 enzymes; and (3) compare the *C*_0_/*D* ratio between two groups of children who took oral solutions or ER tablets of VPA.

## Materials and Methods

### Study Patients and Data Collection

**Figure 1:**
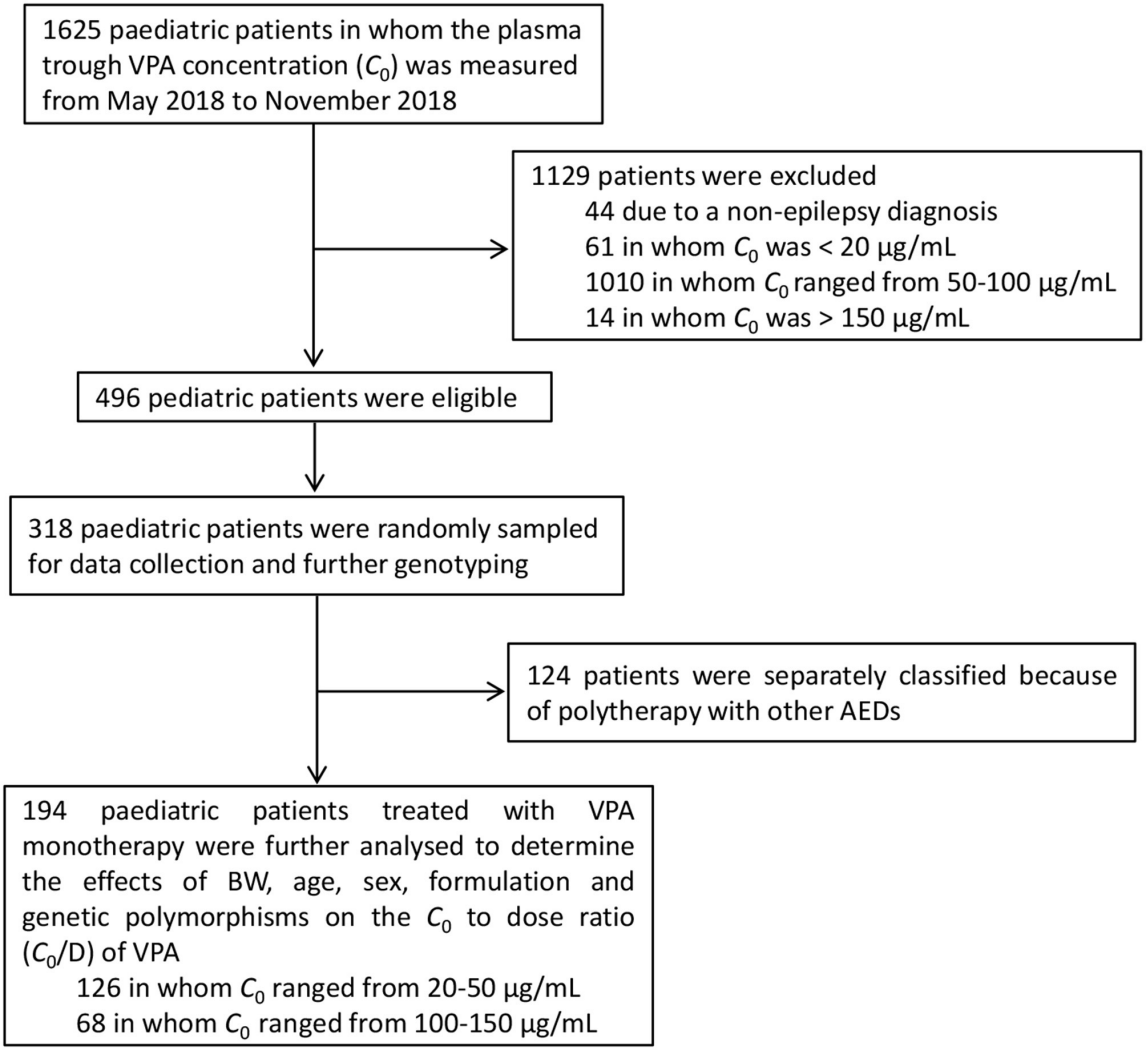
Numbers of pediatric patients who were eligible for inclusion in the study.

This single-center, retrospective cohort study was performed using data from 194 pediatric patients collected between May 2018 and November 2018 at the Children's Hospital of Nanjing Medical University. The study protocol was approved by the hospital ethics committee (protocol number 201902055-1). All of these patients had been diagnosed with epilepsy based on their seizure history, electroencephalogram (EEG), and biochemical laboratory tests. All patients had normal liver and kidney function. The flow diagram of the study cohort is shown in Figure 1. Patients with VAP levels of 50–100 μg/ml (*n* = 1,010) were excluded from the study because most pediatric patients achieve the desired effect in the therapeutic range. Patients with plasma concentration <20 μg/ml (*n* = 60) or >150 μg/ml (*n* = 14) were also excluded for the concerns of non-compliance and the possibility of inaccurate lab results, respectively, which would require further investigation. This study included pediatric patients whose VPA levels were 20–50 and 100–150 μg/ml to examine the effects of variable factors on the *C*_0_/*D* ratio. Patients who had polytherapy with other antiepileptic drugs were excluded. Data collected from the electronic medical records system included basic demographics, laboratory and radiology reports, procedures, medication records, BW (kg), and diagnoses. Based on the dosage forms, patients were divided into the following two groups: patients who took the VPA oral solutions (oral solution group) and patients who received VPA sustained-release (SR) tablets (SR tablet group).

### VPA Monotherapy Protocol

Pediatric patients started VPA oral solutions at a dose of 20 mg/kg/day and the dose was increased to 40 mg/kg/day as tolerated and guided by TDM in patients with severe cases. VPA SR tablets are suitable for pediatric patients aged >6 years old. The oral solution is for individuals who are not willing or unable to swallow the tablets. In the VAP SR tablet group, the initial dose was 10 to 15 mg/kg/day, increased by 5–10 mg/kg/day at weekly intervals until seizures are controlled or side effects preclude further increase.

### Measurement of Plasma VPA Trough Concentration

VPA oral solution or SR tablet administration was maintained for more than five half-lives (at least 3 days) to ensure that blood sampling was performed under steady-state conditions. Peripheral venous blood samples (2 ml) were collected 30 min before the next scheduled dose. Blood was collected into tubes containing the anticoagulant EDTA, and plasma was separated by centrifugation at 4,000 rpm for 8 min at room temperature. The plasma trough concentration of VPA was determined using an automated enzyme immunoassay analyzer (SIEMENS, Munich, Germany). Calibration curves with a range of 1–150 μg/ml and quality control samples with a deviation of ± 15% were applied to ensure the accuracy and precision of the method.

### Definition of the *C*_0_/*D* Ratio

The plasma trough concentration of VPA is abbreviated as *C*_0_ (μg/ml). We defined dose-adjusted plasma trough concentration (*C*_0_/*D*) to indicate the change in the plasma trough concentration of VPA (μg/ml) due to unit dose (mg/kg) administered within 24 h.

### Genotype Analysis

Genomic DNA samples were isolated from peripheral blood samples treating with the anticoagulant EDTA using a DNA extraction kit according to the manufacturer's instructions (BioTeKe Corporation, Wuxi, China). Twenty-six single nucleotide polymorphisms (SNPs) located in genes encoding *CYP2C9*, acylpeptide hydrolase (*APEH*), *UGT1A*, and *UGT2B7* proteins involved in VPA metabolism were genotyped using the MassARRAY System (Agena Bioscience, Inc., San Diego, California). Polymerase chain reaction (PCR) assays and extension primers for these SNPs were designed using the MassARRAY design software (Version 3.1). SNPs were genotyped using the iPLEX Gold assay (Agena Bioscience, Inc., San Diego, California, USA), based on multiplex PCR, followed by a single base primer extension reaction. The masses of the primer extension products correlating with the genotype were determined using matrix-assisted laser desorption/ionization time-of flight (MALDI-TOF) mass spectrometry. Final genotypes were called using the MassARRAY Typer (version 4.0). Five well-studied SNPs (i.e., *UGT2B7* G211T, C802T, C161T, T125C, and CYP2C9^*^3 A1075C) were also included in our study. The mean call rate across the 26 SNPs was 95%, ranging from 90 to 96%. The primer details for genotyping of the 26 SNPs are summarized in Supplementary Table 1. For the quality of genotyping, samples from 10% of all patients were measured repeatedly, and the results were acceptable.

### Statistical Analysis

Statistical analyses were performed using SPSS 22.0 statistical software (IBM, Armonk, USA), and the figures were drawn by using GraphPad Prism 5 software (GraphPad Software, San Diego, USA). The continuous data are presented as means ± SD (standard deviation SD) if they were normally distributed, or expressed as medians and interquartile ranges if not normally distributed. Numbers and percentages were reported for categorical variables. A multiple regression model of the VPA *C*_0_/*D* ratio was established through backward variable screening to explore the factors that affect the plasma *C*_0_. The relationships between BW, age, and the *C*_0_/*D* ratio of VPA were tested by calculating Spearman correlation's correlation coefficients. The Mann–Whitney *U*-test or Kruskal–Wallis *H*-test was used to assess the relationship between the *C*_0_/*D* ratio of VPA and genotype. The allele and genotype frequencies of the *CYP2C9, UGT2B7, UGT1A*, and *APEH* polymorphisms were assessed for deviation from Hardy–Weinberg equilibrium (HWE) using the chi-square test. A *P* > 0.05 was applied for Hardy–Weinberg equilibrium. *P* < 0.05 was considered statistically significant.

## Results

### Characteristics of the Enrolled Pediatric Patients

One hundred ninety-four children with epilepsy (128 males and 66 females) taking VPA monotherapy were enrolled in the present study. The mean age was about 5 years, ranging from < 1 to 14 years. The BW ranged from 8.5 to 74 kg, with a mean value of 23.4 kg. One hundred thirty-five patients received the VPA oral solutions and the other 59 children took SR tablets.

### Effects of BW and Formulations on the *C*_0_/*D* Ratio of VPA

**Figure 2:**
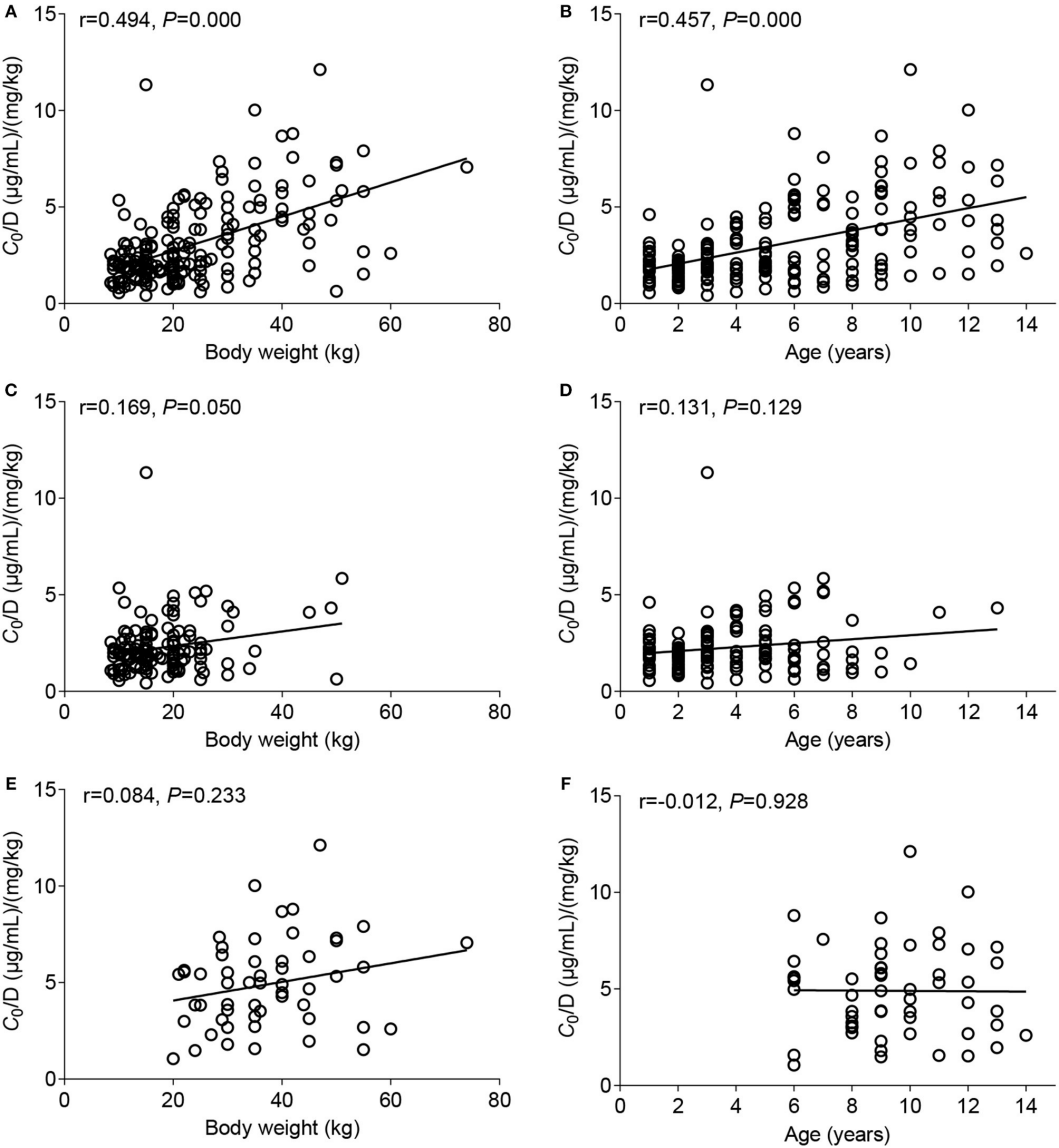
Correlation between the *C*_0_/*D* ratio and body weight (BW) (left column) or age (right column). **(A,B)** Data from patients who took the VPA oral solution and patients who were administered the VPA SR tablets were combined, **(C,D)** analysis of data from patients who took the VPA oral solution, and **(E,F)** analysis of data from patients who took VPA SR tablets.

**Figure 3:**
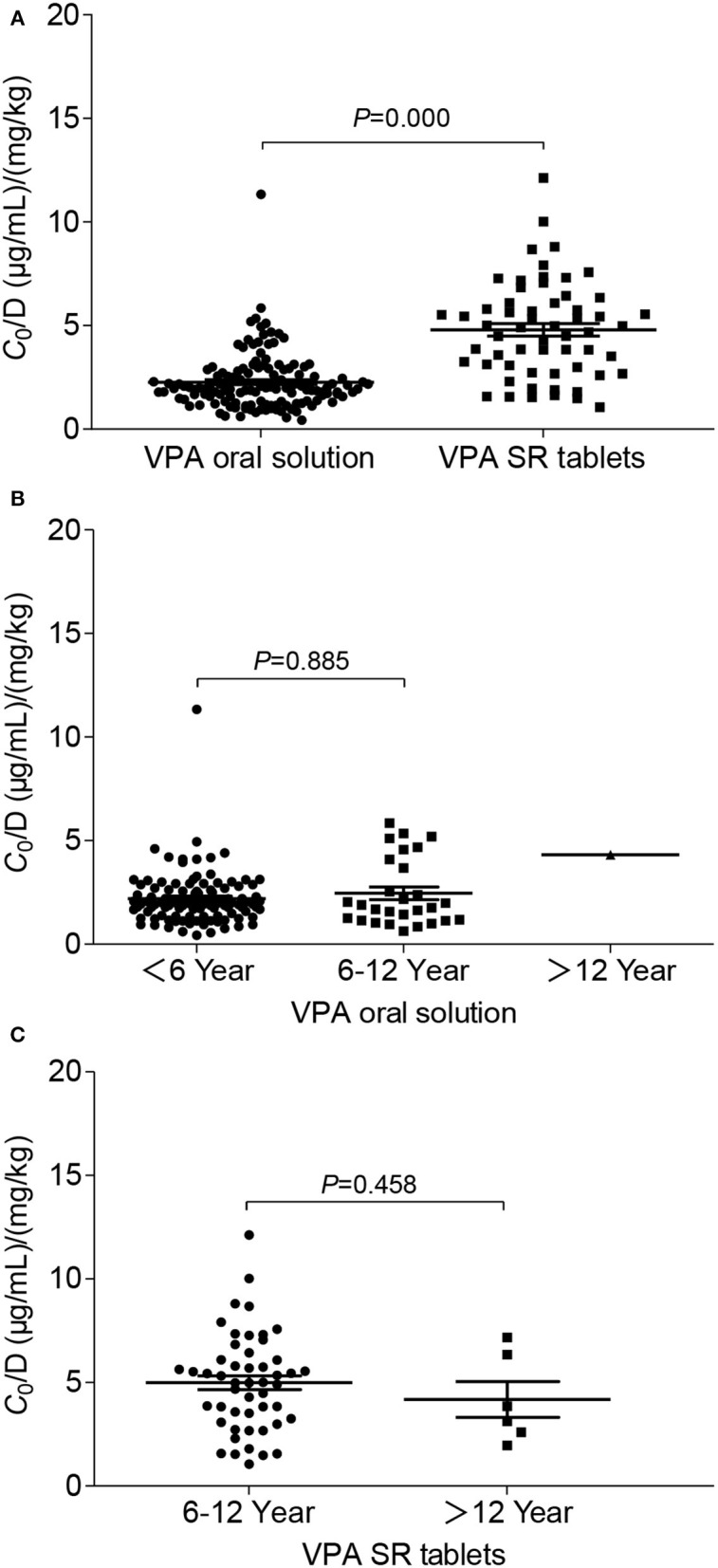
Comparison of the *C*_0_/*D* ratio between patients in different groups: **(A)** VPA oral solution group (*n* = 135) compared with the SR tablet group (*n* = 59); **(B)** age-related effects on the *C*_0_/*D* ratio of VPA in patients who were administered the VPA oral solution, namely the ≤ 6-year-old group (*n* = 106), 6–12-year-old group (*n* = 28), and >12-year-old group (*n* = 1); **(C)** age-related effects on the *C*_0_/*D* ratio of VPA in patients who took VPA SR tablets, namely the 6–12-year-old group (*n* = 50) and the >12-year-old group (*n* = 6).

The potential factors influencing the *C*_0_/D ratio of VPA were evaluated using a linear regression model. BW and the formulation were two major factors that significantly influenced the *C*_0_/*D* ratio (*P* = 0.000) (**Table 3**). Spearman's correlation analysis showed positive correlations of BW and age with the *C*_0_/*D* ratio (Figures 2A,B). However, the positive correlation disappeared when the patients were separated into VPA oral solution group (Figures 2C,D) and VPA SR tablet group (Figures 2E,F). Our subsequent analysis revealed a significant increase in the average *C*_0_/*D* ratio of 2.11-fold in children who took VPA SR tablets compared with children who received VPA oral solutions (*P* = 0.000) (Figure 3A). However, no other significant differences were observed in the different age groups (<6, 6–12, and >12 years old) (Figures 3B,C).

### Effect of Genetic Polymorphisms on the *C*_0_/*D* Ratio of VPA

**Figure 4:**
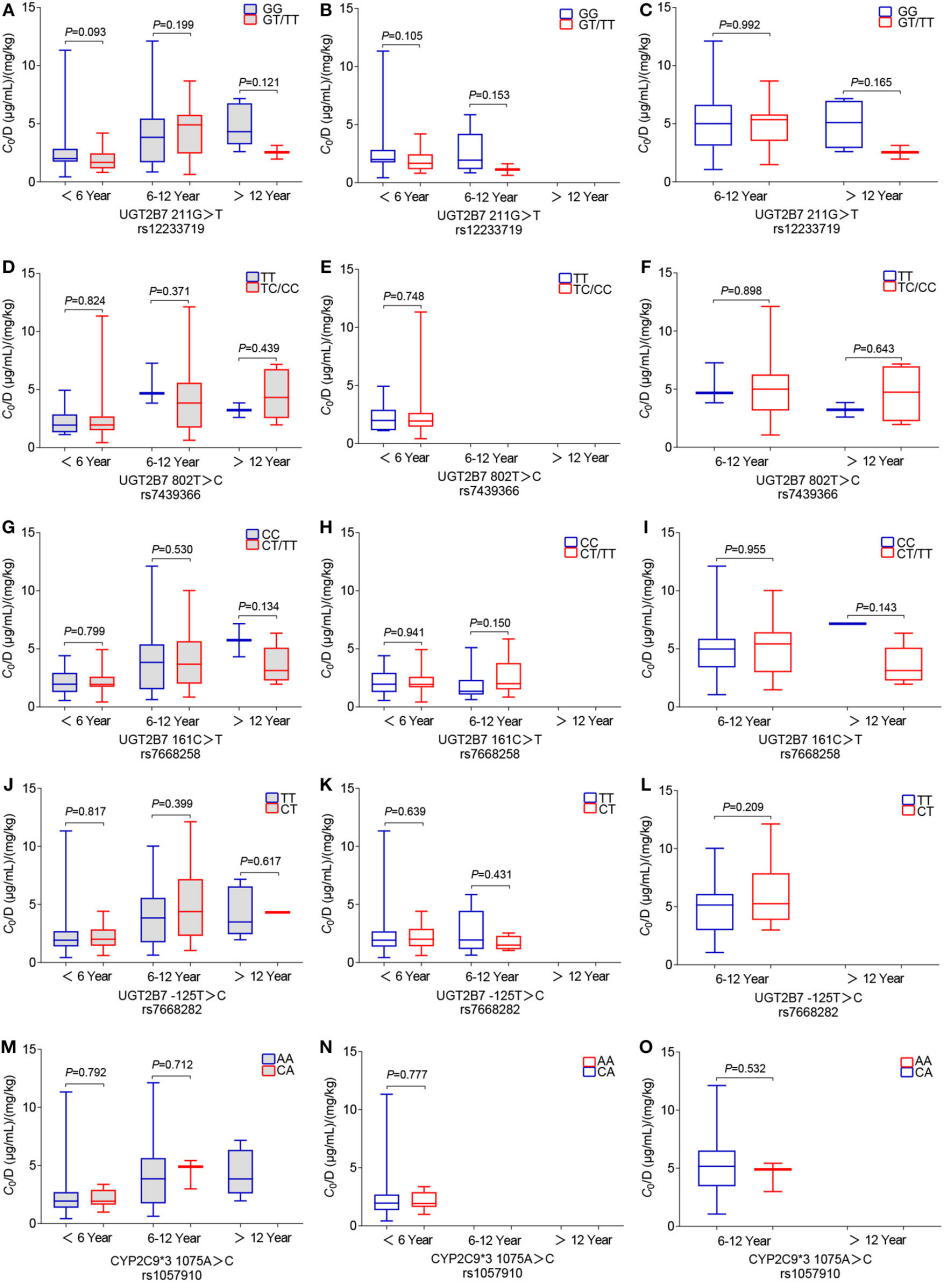
Effects of genetic polymorphisms on the *C*_0_/*D* ratio in different pediatric patient groups: **(A–C)** wild-type and mutant genotypes for *UGT2B7* 211G>T rs1057910, **(D–F)** *UGT2B7* 802T>C rs12233719, **(G–I)** *UGT2B7* 161C>T rs7439366, **(J–L)** *UGT2B7* 125T>C rs7668258, and **(M–O)** CYP2C9*3 1075A>C rs7668282. Here, **(A,D,G,J,M)** show data from the total of 194 patients who were administered the VPA oral solution or SR tablets; **(B,E,H,K,N)** show the data from 135 patients who were administered the VPA oral solution; and **(C,F,I,L,O)** show data from 59 patients who were administered the VPA SR tablets.

Twenty-six SNPs were genotyped for 194 children with epilepsy. The genetic variant rs61764030 failed to be genotyped. Minor allele frequencies (MAFs) of the other nine SNPs (i.e., rs113010112, rs140766748, rs145084767, rs145725367, rs147761911, rs183802414, rs28898619, rs4663870, and rs72551330) were < 0.01 and excluded from further analysis. The other 16 SNPs (rs1057910, rs1131095, rs12233719, rs144486213, rs2070959, rs28898617, rs3816877, rs3821242, rs45549435, rs45615240, rs45625338, rs6759892, rs7439366, rs7668258, rs7668282, and rs8330) were consistent with HWE (*P* > 0.05) (Table 1) and were subsequently analyzed in the 194 patients who took VPA monotherapy. However, no significant association between genetic variants and variable *C*_0_/*D* ratio was detected (*n* = 194) (Table 2), even for the five well-studied SNPs (Figure 4). A significant association between the *C*_0_/*D* ratio and *UGT1A6/9* Del>A (rs144486213) was observed in the VPA oral solution group, but not in the VPA SR tablet group (Table 2).

**Table 1:** The results of the Hardy–Weinberg equilibrium test.

**Gene**	**SNPs**	**Genotype**	**Sample size (*N*)**	**Hardy–weinberg equilibrium**
				***P* value**
CYP2C9^*^3	rs1057910	AA	181	0.683
		CA	11	
*APEH*	rs1131095	TT	166	0.987
		CT	26	
		CC	1	
*APEH*	rs3816877	CC	171	0.423
		CT	21	
UGT1A3^*^3	rs3821242	TT	103	0.147
		TC	71	
		CC	20	
UGT1A3^*^4	rs45625338	CC	166	0.279
		TC	28	
UGT1A3^*^5	rs28898617	AA	170	0.379
		AG	23	
UGT1A6^*^2	rs2070959	AA	117	0.862
		GA	66	
		GG	10	
UGT1A6^*^2	rs6759892	TT	113	0.843
		GT	70	
		GG	10	
*UGT1A6*	rs45549435	DEL	10	0.755
		AGGAG.DEL	71	
		AGGAG	111	
*UGT1A6*	rs45615240	TT	112	0.772
		CT	71	
		CC	10	
*UGT1A6/9*	rs144486213	DEL.DEL	7	0.244
		DEL.A	47	
		AA	138	
*UGT1A1/3/4/6/9*	rs8330	CC	151	0.298
		CG	36	
		GG	4	
UGT2B7^*^2	rs7439366	TT	13	0.317
		TC	84	
		CC	94	
UGT2B7^*^3	rs12233719	GG	137	0.806
		GT	49	
		TT	5	
*UGT2B7*	rs7668258	CC	93	0.757
		CT	73	
		TT	16	
*UGT2B7*	rs7668282	TT	158	0.191
		CT	33	

**Table 2:** Effects of 16 tested SNPs on *C*_0_/*D* ratio in children who took VPA solutions or VPA SR tablets.

**Gene**	**SNPs**	**Genotype**	**Sample size (*****N*****)**			***C*_**0**_/D (μg/ml)/(mg/kg)**	***P*** **value**		
			**Total** ***n* = 194**	**Oral solutions**	**SR tablets**		**Total** ***n* = 194**	**Oral solutions**	**SR tablets**
CYP2C9^*^3	rs1057910	AA	181	126	55	2.20 (1.63–4.14)	0.927	0.814	0.712
		CA	11	8	3	2.56 (1.80–3.38)			
*APEH*	rs1131095	TT	166	110	56	2.34 (1.62–4.30)	0.746	0.73	0.809
		CT	26	23	3	2.23 (1.77–3.12)			
		CC	1	1	0	1.87			
*APEH*	rs3816877	CC	171	118	53	2.29 (1.63–4.09)	0.884	0.902	0.58
		CT	21	16	5	1.99 (1.64–4.40)			
*UGT1A6/9*	rs144486213	DEL.DEL	7	4	3	1.63 (1.08–5.45)	0.379	0.032	0.507
		DEL.A	47	31	16	2.02 (1.36–4.29)			
		AA	138	99	39	2.38 (1.75–4.09)			
UGT1A6^*^2	rs2070959	AA	117	75	42	2.60 (1.65–4.53)	0.404	0.727	0.583
		GA	66	51	15	2.12 (1.50–3.28)			
		GG	10	8	2	2.31 (1.87–5.04)			
UGT1A3^*^5	rs28898617	AA	170	121	49	2.24 (1.68–3.89)	0.841	0.268	0.446
		AG	23	14	9	2.27 (1.35–5.43)			
UGT1A3^*^3	rs3821242	TT	103	76	27	2.20 (1.74–4.11)	0.281	0.052	0.272
		TC	71	45	26	2.60 (1.72–3.87)			
		CC	20	14	6	1.70 (1.12–4.23)			
*UGT1A6*	rs45549435	DEL	10	8	2	2.31 (1.87–5.04)	0.36	0.722	0.583
		AGGAG.DEL	71	56	15	2.08 (1.53–3.38)			
		AGGAG	111	70	41	2.60 (1.63–4.49)			
*UGT1A6*	rs45615240	TT	112	71	41	2.58 (1.64–4.55)	0.341	0.734	0.561
		CT	71	56	15	2.08 (1.53–3.38)			
		CC	10	8	2	2.31 (1.87–5.04)			
UGT1A3^*^4	rs45625338	CC	166	115	51	2.18 (1.58–4.09)	0.158	0.059	0.894
		TC	28	20	8	3.02 (2.00–4.16)			
UGT1A6^*^2	rs6759892	TT	113	71	42	2.60 (1.65–4.53)	0.295	0.72	0.583
		GT	70	55	15	2.06 (1.50–3.28)			
		GG	10	8	2	2.31 (1.87–5.04)			
*UGT1A1/3/4/6/9*	rs8330	CC	151	106	45	2.18 (1.57–3.87)	0.223	0.238	0.794
		CG	36	27	9	2.45 (1.81–4.51)			
		GG	4	1	3	5.19 (2.48–5.73)			
UGT2B7^*^3	rs12233719	GG	137	101	36	2.30 (1.76–4.10)	0.252	0.12	0.81
		GT	49	31	18	2.13 (1.44–3.55)			
		TT	5	2	3	4.20 (2.48–5.77)			
UGT2B7^*^2	rs7439366	TT	13	7	6	2.70 (1.84–4.26)	0.339	0.454	0.527
		TC	84	57	27	2.34 (1.79–4.44)			
		CC	94	70	24	2.14 (1.34–3.85)			
*UGT2B7*	rs7668258	CC	93	70	23	2.07 (1.34–3.84)	0.341	0.609	0.743
		CT	73	50	23	2.29 (1.79–3.89)			
		TT	16	9	7	2.81 (1.82–4.53)			
*UGT2B7*	rs7668282	TT	158	112	46	2.18 (1.66–4.09)	0.462	0.845	0.288
		CT	33	22	11	2.55 (1.60–4.36)			

## Discussion

In the present study, potential genetic and non-genetic factors contributing to the variable *C*_0_/*D* ratio of VPA in pediatric patients with epilepsy were evaluated. Notably, the dosage form, rather than BW, age, or genetic polymorphisms, became the major factor affecting plasma levels of VPA. According to anecdotal reports or retrospective studies, some patients with epilepsy experienced seizures after switching to different formulations or generic products (21, 22). However, a recent randomized trial comparing two generic versions of lamotrigine showed bioequivalence and no significant differences in seizure control (23). In clinical practice, product consistency is recommended because comparative bioequivalence data are incomplete for every AED (24). The findings reported in the present study support the aforementioned advice, and the monitoring of VPA concentrations should be considered in pediatric patients when the dosage forms of valproate are switched.

BW and age were positively correlated with the *C*_0_/*D* ratio (Figures 2A,B), consistent with previous studies (7). However, a positive correlation did not exist when the patients were divided into two groups, receiving either VPA oral solutions (Figures 2C,D) or SR tablets (Figures 2E,F). The multiple linear regression analysis also indicated that the dosage form was a major factor affecting the *C*_0_/*D* ratio (*P* = 0.000) (Table 3). Thus, the dosage form, rather than BW or age, was the key factor influencing VPA *C*_0_/*D* ratio in pediatric patients.

**Table 3:** Results of multiple linear regression analysis (*n* = 194).

**Factors**	**Unstandardized coefficient**		**Standardized coefficient**	***t***	***P* value**
	***B***	**SE**	**Beta**		
Constant	−0.378	0.549		−0.688	0.492
Age	−0.071	0.073	−0.119	−0.975	0.331
Sex	0.035	0.260	0.008	0.135	0.893
Body weight	0.061	0.020	0.371	3.118	0.002
Dosage form	1.771	0.378	0.394	4.684	0.000
**Backward method**					
Constant	−0.223	0.360		−0.619	0.536
Body weight	0.047	0.013	0.285	3.603	0.000
Dosage form	1.649	0.356	0.367	4.636	0.000

Currently, oral solutions and SR tablets are the two most common dosage forms of valproate for pediatric patients with epilepsy. In the early 1990s, Imaizumi and colleagues compared the clinical effectiveness and pharmacokinetic features between a conventional VPA preparation (C-VPA) three times daily and a slow-release granule preparation (SR-VPA) twice-daily administration in Japanese pediatric patients aged from 1 to 16 years. This early comparative study observed better clinical control, a higher steady-state minimum concentration (*C*_min_, i.e., *C*_0_), a lower maximum concentration, and less diurnal fluctuations in the SR-VAPA group than in the C-VPA group after treatment with the same daily dosage (25). The higher *C*_min_ in the modified formulation group was similar to our findings. The authors hypothesized that food might decrease or increase VPA absorption from the C-VPA or the SR-VPA, respectively (25). In a randomized trial, Kernitsky et al. concluded that the total daily dose for patients taking the ER formulation may need to be 8–20% higher when switching from the delayed-release formulation (26). However, Verrotti et al. evaluated the overall effects of the abrupt switch from the solution to VPA modified-release granules administered at identical dosages in children with epilepsy, and no significant differences were observed in plasma VPA levels (27). The conclusions of these previous studies are inconsistent, partially due to the small sample size and comparisons between different drugs and formulations. In the present study, the *C*_0_/*D* ratio of pediatric patients who took VPA SR tablet monotherapy was significantly increased by 2.11-fold compared with patients who took the oral solutions (*P* = 0.000). In fact, direct comparisons of the PK of IR and ER formulations have found that most ER formulations have a lower fluctuation index than the IR versions (e.g., oral solution), and the dose-normalized ER formulations may not be bioequivalent to their IR counterparts (20). Thus, switching between oral solutions and SR tablets should be performed cautiously. If formulation switching is necessary, daily dose adjustment should be considered, and the plasma trough level, seizure-control effect, and adverse drug reaction should also be monitored very closely.

Another aim of this study was to assess the effects of genetic variations on variable plasma levels of VPA. The UGT2B7^*^3 mutation results in similar or decreased enzymatic activity (28). A meta-analysis confirmed that the UGT2B7^*^3 G211T polymorphism (rs12233719) is associated with the *C*_0_/*D* ratio, which was significantly lower in TT genotype carriers than in GG genotype carriers who took VPA monotherapy in East Asia (10). UGT2B7^*^2 C802T (rs7439366), another well-studied genetic variant, was reported to affect the VPA plasma concentration (12). The enzymatic activity of UGT2B7^*^2 mutation was inconsistent (28). Sun and colleagues reported a much lower adjusted *C*_0_ in almost all Chinese adults carrying a T allele than in adults with the CC genotype (12). However, some other studies did not observe an association between UGT2B7^*^2 T802C genotypes and changes in plasma VPA levels in Chinese children (13) or Greek patients (including children and adults) (29). In addition, more studies have revealed significant effects of the *UGT2B7* C161T (rs7668258) (11, 30) and *UGT2B7* T125C (rs7668282) (31) polymorphisms on the VPA *C*_0_/*D* ratio in Chinese children or adults with epilepsy. However, in the present study, neither of these polymorphisms was associated with the VPA *C*_0_/*D* ratio in children with epilepsy who received VPA monotherapy. In fact, the aforementioned studies did not mention dosage forms (11, 30, 32) or perform a separate analysis of those data (11, 12). In our study, the age-related *C*_0_/*D* ratio was significantly associated with *UGT2B7* G211T, and T802C polymorphisms in patients who were administered VPA oral solutions, rather than VPA SR tablets, along with other antiepileptic drugs (data not shown). Notably, the association disappeared when we performed the analysis only in the VPA monotherapy subgroup (Figure 4). Moreover, a significant association between the *C*_0_/*D* ratio and *UGT1A6/9* Del>A (rs144486213) polymorphism was only observed in the VPA oral solution group and not in the VPA SR tablet group (Table 2).

The *CYP2C9* genotype (CYP2C9^*^1/^*^3) was reported to be a poor VPA metabolizer and resulted in a higher VPA level than CYP2C9^*^1/^*^1 in children (*n* = 50, Caucasian) (16) or the northern Han Chinese population (*n* = 179) (18). In the present study, no statistically significant difference in the *C*_0_/*D* ratio was observed among pediatric patients who were CYP2C9^*^3 1075 AA and 1075 CA carriers (Figures 4M–O). Consistent with our findings, CYP2C9^*^3 does not exert an obvious effect on Chinese pediatric patients with epilepsy, or Norwegian adults (8, 33). The MAF of CYP2C9^*^3 (rs1057910) varies in different ethnic groups, i.e., 0.0688, 0.0126, 0.0338, and 0.1131 in European, African, East, and South Asian populations, respectively (34). Even in populations residing nearby, the frequencies of CYP2C9^*^1/^*^1 (69.3–99.1%) and ^*^1/^*^3 (2.3–20.1%) genotypes in most Southeast and East Asian populations display a wide range (35).

In addition, the ontogeny of the aforementioned metabolizing enzymes such as proteins in the UGT2B family contributes to the variable enzyme expression and activity, thereby potentially affecting the metabolism of VPA. Hepatic expression of enzymes in the UGT2B subfamily is characterized by a first phase of *de novo* expression after 20 weeks of gestation in the fetal liver, and the levels measured during fetal development account for 10% of adult levels (36, 37). Afterwards, a second phase of differential upregulation of individual isoforms occurs after 2 years of age (37). The estimated age at which 50% of the adult protein abundance is observed for *UGT2B7* is between 2.6 and 10.3 years, and its microsomal protein abundance increase by 8-fold from neonates to adults (38). In the current study, age was positively correlated with the *C*_0_/*D* ratio (Figure 2B), but this correlation disappeared after patients were analyzed based on the valproate dosage forms (Figures 2D,F).

Collectively, the differences in the findings among these studies might be due to the race or age of the enrolled patients, the stratification of different subgroups, or the sample size. Therefore, the clinical data should be interpreted with caution, particularly when those data are not analyzed separately.

However, our study has several limitations. This retrospective single-center study had a small sample size. The inclusion of too few patients in a study cohort increases the risk that a significant change will not be detected, even if a change exists. In this study, genetic *UGT2B7* polymorphisms did not affect the *C*_0_/*D* ratio. However, the findings should not be described as conclusive because of the small sample size. Second, data on the *C*_0_/*D* ratio of VPA from individual patients who switched formulations between oral solutions and SR tablets was unavailable to evaluate intrapatient changes. In addition, sample collection and subsequent assays are important to provide opportune concentration data. Trough levels were collected 30 min before the next scheduled dose in the morning for those inpatients. For those outpatients (*n* = 185), the timing of blood sampling was monitored by physicians and pharmacists. Trough levels were collected in the early morning (7:00–7:30 A.M.) or late afternoon (4:30–5:00 P.M.) given the limitations on the operational hours of the laboratory and the clinic. The time of blood sampling probably affects the real plasma VPA concentration.

## Conclusions

We concluded that dosage forms of sodium valproate, but not BW, age, or genetic polymorphisms, significantly affected the VPA *C*_0_/*D* ratios in pediatric patients with epilepsy in our present study. Based on our findings, switching between solutions and SR tablets should be performed cautiously. More importantly, total daily dose adjustment should be considered, and the plasma *C*_0_, seizure-control effect, and adverse drug reaction should also be monitored very closely.

## Data Availability Statement

The original contributions presented in the study are included in the article/Supplementary Materials, further inquiries can be directed to the corresponding author/s.

## Ethics Statement

The studies involving human participants were reviewed and approved by Children's Hospital of Nanjing Medical University. Written informed consent to participate in this study was provided by the participants' legal guardian/next of kin.

## Author Contributions

Z-YX, H-LG, FC, and JX are the principal investigators of the study and the primary authors of the manuscripts. Z-YX, H-LG, and FC collected and analyzed the data. LL, XJ, and Z-JX assisted in performing the study and data analysis. J-CQ, X-PL, and X-SD assisted with the design, performance of the human experiments, and the writing of the paper. MZ critically reviewed and revised the manuscript. All authors have read and approved the final manuscript.

## Conflict of Interest

The authors declare that the research was conducted in the absence of any commercial or financial relationships that could be construed as a potential conflict of interest.

## Abbreviations

AEDs: antiepileptic drugs
APEH: acylpeptide hydrolase
BW: body weight
*C*_0_: plasma trough concentration
*C*_0_/*D* ratio: dose-adjusted plasma trough *C*_0_
C-VPA: conventional valproic acid preparation
CYPs: cytochrome P450
ER: extended-release
EEG: electroencephalogram
HWE: Hardy–Weinberg equilibrium
IR: immediate-release
MAF: minor allele frequency
PCR: polymerase chain reaction
PK: pharmacokinetics
SR: sustained-release
SR-VPA: slow-release granule preparation
SNPs: single-nucleotide polymorphisms
TDM: therapeutic drug monitoring
UGTs: UDP-glucuronosyltransferases
VPA: valproic acid
VPA oral solution: sodium valproate oral solution
VPA SR tablets: sodium valproate sustained-release tablets.
