# Supplementary material for: Genetic and Non-genetic Factors Contributing to the Significant Variation in the Plasma Trough Concentration-to-Dose Ratio of Valproic Acid in Children With Epilepsy
Source: Front Pediatr. 2021 Jan 20;8:599044. doi: 10.3389/fped.2020.599044 (PMC7855978; doi:10.3389/fped.2020.599044)
Supplement: Supplementary file 1 [file Table_1.DOCX]

**Genetic and non-genetic factors contributing to the significant variation in the *C*_0_/D ratio of valproic acid in children with epilepsy**

Ze-Yue Xu 1,2†‡, Hong-Li Guo 1†, Ling Li1,2‡, Min Zhang 3, Xia Jing 1, Ze-Jun Xu 1, Jin-Chun Qiu 1, Xiao-Peng Lu 4, Xuan-Sheng Ding 2, Feng Chen 1, *, Jing Xu 1, *

*1 Department of Pharmacy, Children’s Hospital of Nanjing Medical University, Nanjing, China,*

*2 School of Basic Medicine and Clinical Pharmacy, China Pharmaceutical University, Nanjing, China,*

*3 Department of Pharmacy, Boston Medical Center, Boston, MA, United States,*

*4 Department of Neurology, Children’s Hospital of Nanjing Medical University, Nanjing, China*

^⁎^ Corresponding authors.

*E-mail addresses*: [cy.chen508@gmail.com](mailto:cy.chen508@gmail.com) (F. Chen), [njxujing@163.com](mailto:njxujing@163.com) (J. Xu).

^†^These authors contributed equally to this work.

^‡^Visiting graduate student from China Pharmaceutical University.

**----Supplemental data----**

Supplemental Table 1 Genotyping information for 26 selected single-nucleotide polymorphisms (SNP)

| **Gene** | **SNP-ID** | **Variants** | **1st-PCRP** | **2nd-PCRP** | **Amplification length(bp)** | **Extension sequence primer** |
| --- | --- | --- | --- | --- | --- | --- |
| *CYP2C9*3* | rs1057910 | 1075A>C | ACGTTGGATGTGTCACAGGTCACTGCATGG | ACGTTGGATGCTACACAGATGCTGTGGTGC | 103 | tgggtCACGAGGTCCAGAGATAC |
| *APEH* | rs1131095 | T>C | ACGTTGGATGTCAGCCACTTACCACACTTC | ACGTTGGATGGACTGAGTGTCTGTCTCGTG | 100 | AGGTCAGCCCTGTATTA |
| *APEH* | rs3816877 | C>T | ACGTTGGATGTGGACATCCTTCACATCCTG | ACGTTGGATGGCCCTCAGTGAACTATCGTG | 116 | cctaAGTGAACTATCGTGGCTCCA |
| *UGT1A1/3/4/6/9* | rs8330 | 12712G>C | ACGTTGGATGCTTTAAACACACAAGGTGGC | ACGTTGGATGTTGGCTTCTGCAGATGGTTG | 115 | TCAGTCCTCATCTCTGTC |
| *UGT1A3* | rs28898617 | 17A>G | ACGTTGGATGGACACTGAGGAGAAGCAGCA | ACGTTGGATGTCCGTGTCTTCTGCTGAGAT | 104 | cAGATGGCCACAGGACTCC |
| *UGT1A3*3* | rs3821242 | 31T>C | ACGTTGGATGTCCGTGTCTTCTGCTGAGAT | ACGTTGGATGGACACTGAGGAGAAGCAGCA | 104 | ccaaAGCAGTCCTGTGGCCAGCC |
| *UGT1A3*4* | rs45625338 | 133C>T | ACGTTGGATGTTGATGGCAGCCACTGGCTC | ACGTTGGATGTGTGCATATTCACCTCTGGG | 119 | acactGAGCTCCCGCAAGACCTCCC |
| *UGT1A3*7* | rs113010112 | 328T>A | ACGTTGGATGGGCCACACTCAACTGTACTT | ACGTTGGATGGACCTATGATAGACCAAAGAC | 118 | AGACATATTGTTCAACATTGC |
| *UGT1A3*8* | rs61764030 | 473C>T | ACGTTGGATGAGGCACCTGAATGCTACTTC | ACGTTGGATGATCGACAGGTACTTAGCCAG | 106 | tTACTTAGCCAGCACTGCC |
| *UGT1A3*9* | rs140766748 | 622A>C | ACGTTGGATGGTAGAGCATGTTCTTGACCC | ACGTTGGATGACACAGTGTCCAAACCCTTC | 116 | caAATTCAGACCACATGACATTC |
| *UGT1A3*11* | rs28898619 | 342G>A | ACGTTGGATGGACCTATGATAGACCAAAGAC | ACGTTGGATGGGCCACACTCAACTGTACTT | 118 | GAAACAGAACATTTTCTGAAGAAAT |
| *UGT1A4*7* | rs183802414 | 271C>T | ACGTTGGATGAAGAACCCTTGAGTGTAGCC | ACGTTGGATGTTTCACCCTGACAGCCTATG | 101 | GACCCAGAAGGAATTTGAT |
| *UGT1A6*7* | rs147761911 | 408G>A | ACGTTGGATGGAGCAGTTAGGAATGATCGC | ACGTTGGATGCGTATGACCAAGAAGAGCTG | 101 | ttAGAAGAGCTGAAGAACC |
| *UGT1A6*2* | rs2070959 | 541A>G | ACGTTGGATGATCTGTGTACCTCTTCAGGG | ACGTTGGATGTGTAGCACCTGGGAATGTAG | 112 | TGGGCTTCTGCTGAATG |
| *UGT1A6* | rs45549435 | -1310->AGGAG | ACGTTGGATGAAGCCTGGTATCTGCTTCTG | ACGTTGGATGCTGGGCCTCAGTTGAGAAAC | 108 | gTGAGAAACCTGAAGCTCAGGAG |
| *UGT1A6* | rs45615240 | -2355T>C | ACGTTGGATGATCCCTAAAGAGTAGCTCCG | ACGTTGGATGAAGTGATACCTGAGGACACC | 102 | GGACACCTTGACTCC |
| *UGT1A6* | rs6759892 | 19T>G | ACGTTGGATGTTAACTCTTTCCAGGATGGC | ACGTTGGATGTGCCCCAAAGTGCTAAGAAG | 102 | ggTGCAGAAATTCTCTGAAATG |
| *UGT1A6/9* | rs144486213 | -/A | ACGTTGGATGATATGTCCCAAGCCTGAGTC | ACGTTGGATGACTTGAGTGTCCAGGCAAAG | 101 | gacatGCAAAGGCCGATTTTTTTTTTT |
| *UGT1A9*2* | rs145084767 | 8G>A | ACGTTGGATGACACACACATAGAGGAAGGG | ACGTTGGATGCTTAGATTCCCAGCTGCTTG | 105 | ccccCAGTTCTCTGATGGCTT |
| *UGT1A9*3* | rs72551330 | 98T>C | ACGTTGGATGGACCTCATGGTGAACCAGTG | ACGTTGGATGTATGTGTGTGTCTGCTGCTG | 114 | gggcGGGAAGCTACTGGTAGTGCCCA |
| *UGT1A9*5* | rs4663870 | 766T>A | ACGTTGGATGGGCAGTTGATACCACCAATG | ACGTTGGATGGTTGTTGCGAACGGACTTTG | 100 | GACTATCCCAAACCCG |
| *UGT2B7* | rs12233719 | 211G>T | ACGTTGGATGGATGAAATTCTCCAACTCAG | ACGTTGGATGCCATTCTTTTTGATCCCAAC | 109 | cTCCCAACAACTCATCC |
| *UGT2B7* | rs7668282 | -125T>C | ACGTTGGATGCCCTTATAAGTCAAAGTACA | ACGTTGGATGGTGTGAACAGATCATTTACC | 115 | CTCAGACTGTTGATTTAATGA |
| *UGT2B7* | rs7668285 | 161C>T | ACGTTGGATGCCCTTATAAGTCAAAGTACA | ACGTTGGATGGTGTGAACAGATCATTTACC | 115 | ccGATCATTTACCTTCATTTGTCTC |
| *UGT2B7*2* | rs7439366 | 802T>G | ACGTTGGATGGCTGACGTATGGCTTATTCG | ACGTTGGATGTGGAGTCCTCCAACAAAATC | 106 | gACATTTGGTAAGAGTGGAT |
| *UGT2B7*4* | rs145725367 | 1192G>A | ACGTTGGATGGGCCTTCATGTGAGCAATGT | ACGTTGGATGCATCTACGAGGCAATCTACC | 108 | ccaacGGGGATTCCATTGTTTGCC |

*Abbreviations: CYP2C9, Cytochrome P450 2C9 gene; APEH, acylpeptide hydrolase gene; UGT1A3, UDP-glucuronosyltransferase 1A3 gene; UGT1A4, UDP-glucuronosyltransferase 1A4 gene; UGT1A6, UDP-glucuronosyltransferase 1A6 gene; UGT1A9, UDP-glucuronosyltransferase 1A9 gene; UGT2B7, UDP-glucuronosyltransferase 2B7 gene.*
